# A task-based approach for Gene Ontology evaluation

**DOI:** 10.1186/2041-1480-4-S1-S4

**Published:** 2013-04-15

**Authors:** Erik L Clarke, Salvatore Loguercio, Benjamin M Good, Andrew I Su

**Affiliations:** 1 The Scripps Research Institute, La Jolla, CA, USA

## Abstract

**Background:**

The Gene Ontology and its associated annotations are critical tools for interpreting lists of genes. Here, we introduce a method for evaluating the Gene Ontology annotations and structure based on the impact they have on gene set enrichment analysis, along with an example implementation. This task-based approach yields quantitative assessments grounded in experimental data and anchored tightly to the primary use of the annotations.

**Results:**

Applied to specific areas of biological interest, our framework allowed us to understand the progress of annotation and structural ontology changes from 2004 to 2012. Our framework was also able to determine that the quality of annotations and structure in the area under test have been improving in their ability to recall underlying biological traits. Furthermore, we were able to distinguish between the impact of changes to the annotation sets and ontology structure.

**Conclusion:**

Our framework and implementation lay the groundwork for a powerful tool in evaluating the usefulness of the Gene Ontology. We demonstrate both the flexibility and the power of this approach in evaluating the current and past state of the Gene Ontology as well as its applicability in developing new methods for creating gene annotations.

## Background

### Introduction

The Gene Ontology [1] (GO) provides a resource for systematically classifying and annotating gene function. The annotations associated with the GO play a critical role in modern biology and cover many organisms. Many of these annotations, especially for human, are the product of manual and automated annotation by the Gene Ontology Annotation (GOA) team at UniProt [2]. For the human genome, over 10,000 GO terms are used to annotate gene function in over 200,000 annotations.

**Figure 1:**
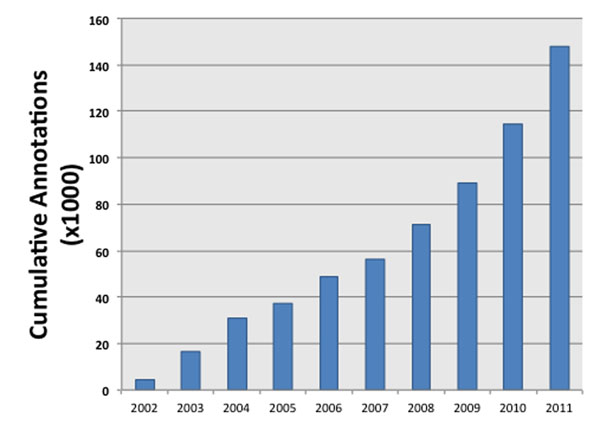
**Cumulative annotations from 2002 through 2011** The increase in individual gene:term annotation pairs from the start of 2002 through the start of 2012. These numbers were created by progressively filtering earlier annotations from the 2012 human annotation file available at [3].

Annotations in GOA come from a variety of sources. Broadly, they either derive from manual curation, or from automatic inference based on pre-existing annotations and resources. Currently over half of the human GO annotations are the result of manual curation as opposed to the automatic electronically inferred annotations (IEAs) [3]. Historically, manually-added annotations are considered to be of higher quality [4] than IEAs, though recent work is challenging this conception [5]. Both sets of annotations are continually revised and expanded based on published advances in the literature and sustained biocuration efforts. The ever-increasing size of the human GO annotation dataset (Figure 1) suggests that the structured representations of gene function are still very much in flux.

Due to the importance of GO annotation in modern biology, significant effort has been expended to assess the quality of these annotations. Measures of annotation completeness, accuracy, and precision are important tools for the GO developers, and are critical if researchers are to use the annotations in real-world applications with confidence. Numerous methods for assessing annotation error rate and accuracy have been developed [6-8]. However, most of these focus on the creation of *ad-hoc* qualitative metrics based on annotation evidence codes and term specificity. Such methods, (e.g. [7]) have significant drawbacks: for one, it is conceivable to construct a random and artificial ontology that would score highly on these metrics without any bearing on the real world. Likewise, measures of accuracy based on term specificity have been called into question [5]. Other approaches that address annotation error rates or accuracy such as [6] and [8] downplay the role of ontology structural quality, and ignore the effect that the ontology structure can have on real-world applications.

An approach that quantified the performance of the ontology at common tasks would allow us to understand the strengths and weaknesses of the ontology and its annotations directly [9]. In this paper, we present an approach that assesses the quality and utility of GO and its annotations through their performance in a common use-case, namely gene-set enrichment analysis.

### A task-based approach

Enrichment analysis is the process of using a collection of gene annotations to determine what terms are “enriched” or over-represented in a set of genes, which are often produced from genome-scale experiments (e.g., gene expression analysis, genome sequencing, etc.). For instance, a cancer researcher, presented with a set of genes implicated in neuroblastoma metastasis, could use enrichment analysis and the GOA to determine what biological processes are most associated with her gene set. This process is among the most common applications of the GO annotations, and is a critical resource for the analysis of genome-scale profiling experiments [10][11][12].

**Figure 2:**
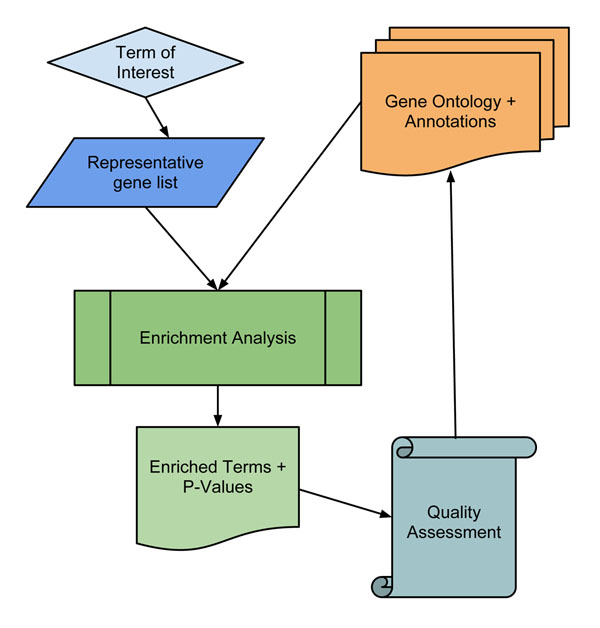
**Analytical framework for evaluation of GO performance** Using our method, the user first identifies a GO term or biological area of interest. He or she then collects experimental data known to be representative of that area, and performs an enrichment analysis with the gene list and appropriate GO annotation sets. By identifying the significance (or lack therof) of the term or terms associated with the area of interest, the user can quantify the performance of the GO and its annotations for this domain.

Our framework uses enrichment analysis to determine the effectiveness of the GO annotations at providing biologically accurate results (Figure 2). This produces a measure of utility based on a real-world application, and helps us understand the strengths and weaknesses of the GO and its annotations. Broadly, our framework consists of the following workflow: First, we select one or more terms to test that are representative of our area of interest. Next, select a dataset that is clearly representative of those terms, and use standard statistical methods to define a list of differentially expressed genes. Conduct an enrichment analysis, using the GO annotations and the chosen dataset, and obtain a list of terms that are significantly enriched among the list of differentially expressed genes. The performance of the GO knowledgebase is related to the significance of the term of interest in the resulting list. Notice that the performance measured in this manuscript focuses on the specific biological context chosen, and not the quality of the GO knowledgebase as a whole. Rather, we provide a framework for evaluating its precision and coverage in specific domains where compelling experimental evidence is available.

### Implementation and testing

To demonstrate the framework, we present an example implementation and apply it towards a selected term of interest. The methodology and results of this experiment are discussed below.

## Results

### Creating the Analytical Framework

Our framework consists of the following steps:

1 Identify term(s) of interest *T*. These may be representative terms of an area of interest or a sample across the GO structure.

2 Collect experimental data that are clearly related to *T.* For example, if *T* was *angiogenesis*, one might use gene expression datasets related to highly angiogenic tissues to derive a list of overexpressed genes.

3 Use standard statistical methods to define a list of differentially expressed genes.

4 Conduct an enrichment analysis on each gene list from (3).

5 From the list of enriched terms resulting from (4), identify the rank and p-value of *T*. The expectation is that *T* will be significantly enriched against the background terms; whether or not that expectation is met, along with *T*’s relative rank and score, indicates the efficacy of *T*’s annotation set.

The framework as presented here is generalizable. For instance, it is up to the user to determine what enrichment method to perform in Step 4. It is also up to the user to determine how to best identify terms of interest and the associated datasets.

One basic use case is to evaluate how well the GO annotations perform at reproducing biological expectations for a dataset. A researcher interested in angiogenesis may collect a series of datasets derived from tumor samples that are known to express traits associated with blood vessel formation, for instance. He or she would then use these datasets in an enrichment analysis with the current version of the GO annotations, and observe how significant the angiogenesis-related terms were in the resulting list of enriched terms. The researcher could compare the rank of angiogenesis terms to other unassociated biological processes to get an idea of how well the GO annotations cover this area. This procedure could be used in any area for which experimental data are available. To test these various analyses, we implemented the framework as a set of Python scripts called the GO Evaluation Suite (GOES). This resource is available as open-source software [13].

### Test 1: Evaluating *current* GO performance on a selected dataset

In our tests, we selected the GO term *angiogenesis* (GO:0001525) as our term of interest. Angiogenesis, the formation of blood vessels, is a well-known trait of a type of brain tumor called glioblastoma multiforme. Glioblastoma multiforme, a high-grade glioma, is unusual for tumors because of its highly vascular nature [14]. We then searched the NCBI Gene Expression Omnibus [15] (GEO) for expression microarray datasets involving glioblastoma and selected one such dataset, GDS1962 [16]. GDS1962 compares general gene expression in various gliomas, including glioblastoma, to non-tumor control samples. The structure of the experiment that produced this dataset and the tissues it tested made us reasonably confident that angiogenesis was a highly significant trait of the glioblastoma samples. We therefore expect that conducting an enrichment analysis using the Biological Process branch of the GO would yield *angiogenesis* as a significant term. Our expectations were validated: an enrichment analysis using the protocol (outlined in more detail in Methods) yielded a p-value of 1.07 x 10^-8^ for the term *angiogenesis.* Even after applying a conservative Bonferroni multiple-testing correction, this enrichment for *angiogenesis* was still significant in GDS1962 (p = 6.3 x 10^-5^).

### Designing a time-based analysis

We can expand this method to assess the changes made to the GO and its annotations over time. Because the GO is constantly changing, it is important to be able to determine if the alterations improve or decrease its performance in common use-cases. To assess the quality of the GO and its annotations over time, we performed the following experiment:

1 Select terms and representative datasets as in Steps 1 and 2 in the original framework.

2 By using the version-control system of the Gene Ontology Consortium [17] retrieve versions of the GO structure for each time point under consideration. For instance, to look at the changes from 2004 - 2012, we would collect nine iterations of the GO, one from each year.

3 Retrieve matching iterations of the GO annotations from [18].

4 For each time point, complete the remaining steps in the original framework. Note the significance of the term(s) of interest in each time point.

5 Plot or otherwise compare the changing significance of the term across the time points.

### Test 2: Evaluating GO performance over time

**Figure 3:**
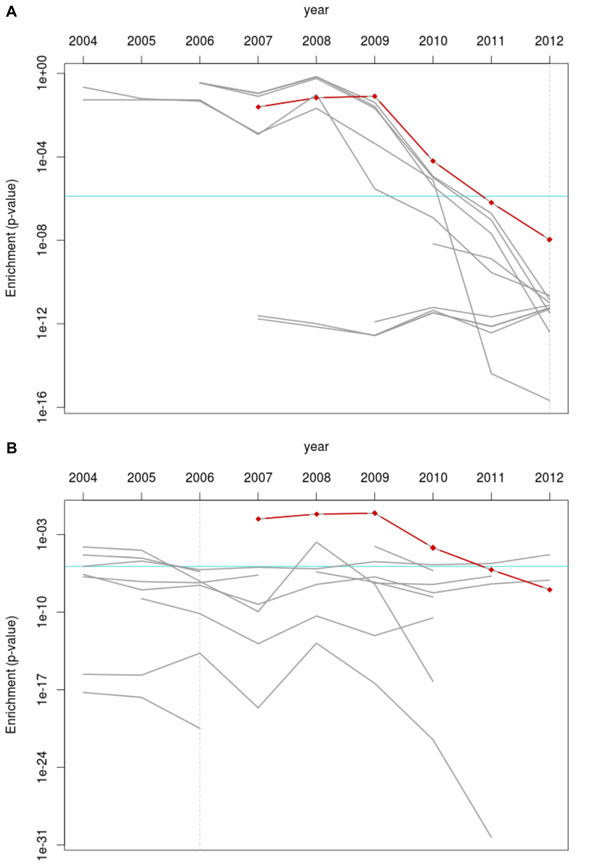
**P-values of *angiogenesis* and ten most significant terms for glioblastoma samples over time A)** The red line shows the change in p-value of the *angiogenesis* term from 0.024 (2007) to 1.07x10^-8^ (2012). The grey lines are the p-values for the ten most significant terms in enrichment analysis from 2012 traced through time (see Table 1). The vertical axis is on a log scale. Some lines start after 2004 or end before 2012 due to filtering in the enrichment analysis (only terms annotated to between 3 and 500 genes). The final p-values have had a Bonferroni multiple-testing correction applied. **B)** The same as A but using the most significant terms from enrichment analysis done with 2006 versions of the ontology and annotations.

We implemented the time-based analysis by sampling the structure of the GO in May of each year from 2004 through 2012 from the GO version control repository and the human GO annotations from concurrent time points in the GOA repository. We produced each term’s gene set by associating that year’s annotations to the terms in the ontology. The rest of the procedure was identical to the previously-described process, and yielded the significance of the term *angiogenesis* for GDS1962 from 2004 to 2012. Figure 3A (red line) shows the changing p-value for *angiogenesis* in the glioblastoma sample during this time. From 2007 to 2012, the p-value of *angiogenesis* decreased by nearly five orders of magnitude (0.024 to 1.07 x 10^-8^).

**Table 1:** Top Ten Biological Process Terms for 2006 and 2012

2006	2012
System development	axon guidance
nervous system development	regulation of synaptic transmission
transmission of nerve impulse	regulation of transmission of nerve impulse
mitotic cell cycle	M phase of mitotic cell cycle
intracellular protein kinase cascade	nuclear division
metal ion transport	mitosis
cell morphogenesis	organelle fission
cytoskeleton organization	regulation of vesicle-mediated transport
regulation of cell cycle	regulation of neurological system process
potassium ion transport	learning or memory

The p-values for 2006 range from 3.15E-21 to 6.70E-7. The p-values for 2012 range from 1.11E-16 to 2.26E-10. The terms are listed in order of p-value.

### Tracking the significance of the most relevant terms over time

Another variation of the framework is the omission of pre-identified terms of interest in favor of observing the most significant terms for a dataset. More specifically, instead of *a priori* identifying a term of interest, we select a gene list of interest and run an enrichment analysis on those genes. We then select the most significant terms and observe how their significance changes as the structure and annotations change (as in the time-based analysis). The benefit of this approach is that it yields a broader picture of the GO evolution by identifying what was reported as being most significant for a dataset in past versions of the GO. It allows researchers to see how recently a term became significant, and which terms have become less relevant.

### Test 3: Behavior of the most significant terms over time

To see if the most significant terms for GDS1962 in 2012 had always been the most significant through the years, we followed the generalized dataset analysis approach described in Methods. First, we selected the top 10 “most significant” terms (i.e. lowest p-values) from an enrichment analysis using up-to-date versions of the ontology and annotations. We then conducted a time-based analysis to track the significance of these terms in past years, shown in Figure 3A (grey lines). Because the study that produced GDS1962 was published in 2006, we also examined how the top terms of an identical enrichment analysis done in 2006 would perform in later years (Figure 3B, grey lines). In these figures, terms that had a p-value of 1 or were annotated to fewer than 3 genes were omitted, resulting in the grey lines beginning only when the terms started appearing. In Figure 3A, we can see that some terms are consistently highly significant from 2007 onwards, while some only appear as recently as 2010. In Figure 3B, the behavior of the top terms in 2006 is inconsistent: while some improve in significance, others become less relevant.

### Comparison of changes to ontology structure and changes to annotations

The Gene Ontology and its annotations are distinct entities. Changes to the ontology structure, such as the addition or clarification of a term, or the movement of a term within the graph, may affect the results of enrichment analyses independently from annotation changes[19]. Our time-based framework can be modified to assess the impact of these different kinds of changes in the following manner.

Before, we combined the ontology structure for a given time point with its contemporary annotations, recreating the resource as it would have been used during that time. By only varying the ontology structure, and using a single set of annotations, we can observe the impact of structural changes on performance. Conversely, we can observe the impact of annotation changes alone by using various annotation sets while using a set ontology structure. We then use these modified resources in the time-based analysis specified above.

### Test 4: Assessing the difference between changes to ontology structure and annotation

**Figure 4:**
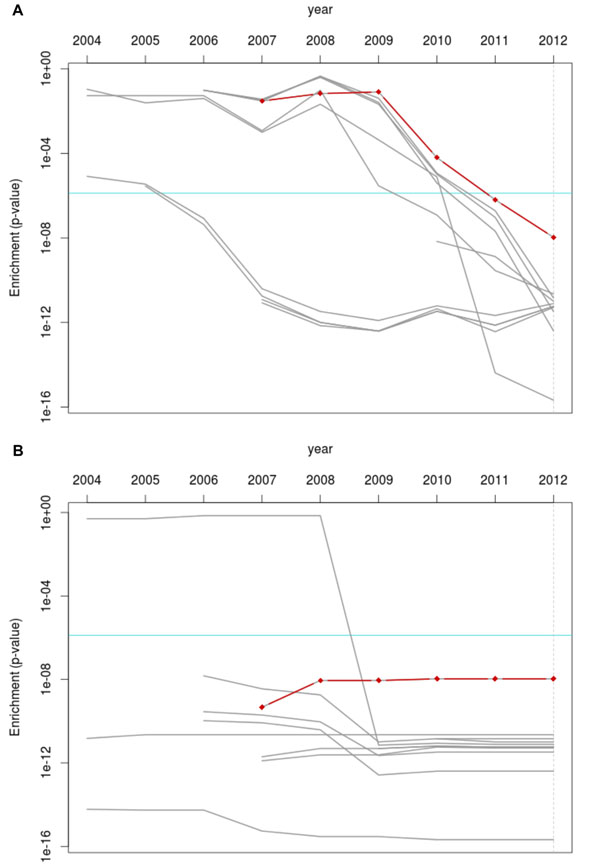
**Effect of changing only the annotations or ontology structure on *angiogenesis* and most significant terms A)** The same analysis as in Figure 3A, but with only the annotations changing over time (the ontology structure used was from 2012). The final p-values have had a Bonferroni multiple-testing correction applied. **B) **The same analysis as Figure 3A, but with only the ontology structure changed (the annotation set used was from 2012).

We created gene sets reflecting ontology structure changes by merging the 2012 annotations with each year’s version of the GO. Similarly, we produced gene sets reflecting annotation changes by merging each year’s annotations with the 2012 GO structure. We then performed an enrichment analysis on each collection of gene sets and looked at the performance of both *angiogenesis* and the previously-identified top 10 most significant terms for glioblastomas in GDS1962. Figure 4 shows the results when considering only changes to the annotations (Fig. 4A) or only changes to the ontology structure (Fig. 4B). Generally, changes to the ontology structure did not have as significant an effect as changes to the annotation sets, as evidenced by the relatively unchanging significance levels in Fig. 4B compared to Fig. 4A.

## Discussion

Here, we presented a task-based framework for GO annotation evaluation and applied it to an angiogenesis-enriched gene list derived from a glioblastoma dataset. We showed how our method can be used to analyze the historical performance of the GO, providing us with an understanding of how the ontology and annotations have changed over time. Our framework also allowed us to separate the effects of changes to the ontology structure from changes to the annotations, and to see how each affects the performance of the GO in a real-world task.

Our implementation of the framework shown here presents us with some noteworthy results. Our term of interest, *angiogenesis*, rose in significance over time, as we had expected. What we had not expected was how recently some of the most significant terms in the 2012 analysis even started appearing in results (a term appeared if it had a p-value of 1 or annotated to more than 3 genes)- some as late as 2010. Other terms show a dramatic increase in p-value from 2010 to 2012. To determine whether changes to the ontology or the annotations were responsible for these trends, we can consider our structure-vs-annotations experiment shown in Figure 4. Here, we see that the graph where only the annotations changed (Fig. 4A) shows the same trends of interest as Fig. 3A, while the graph with only ontology changes lacks these trends. We can safely assert that annotation changes, *in this instance*, are largely responsible for the later dramatic changes. These results only hold for our dataset under test; an interesting follow-up to this would be to identify a GO term with low annotation activity and see if ontology structure changes affect it more strongly.

In Figure 3B, we see that the significance of the most relevant terms in the 2006 enrichment analysis do not consistently improve, and are not especially significant in later years. In fact, Table 1 shows that there is no overlap between these terms and their counterparts in 2012. However, some terms are closely related and have more specific counterparts in 2012, e.g. *transmission of nerve impulse* becomes *regulation of transmission of nerve impulse*.

For this dataset, our results suggest that the GO and its annotations have become more effective at representing the underlying biological facts of the data. We can assert this based on the rising significance of a key tissue phenotype, angiogenesis, and the increasing specificity of its most significant terms. Questions on the overall efficacy of the GO and the human GO annotations are not answered by our implementation.

### Discussion of the framework

These results illustrate the behavior of the GO as applied to a single dataset, which begs the question of whether these results would hold in a more general analysis. The demonstrable flexibility of the framework would allow its use in a large-scale effort where representative terms or areas of interest are selected and tested. For instance, with datasets that are specifically crafted to represent particular traits like cell division or apoptosis, we could determine empirically how accurate and useful the GO and its annotations are at representing biological truths.

On a smaller scale, the framework allows researchers to test modifications to the ontology or annotations in real-time. Let us assume a new method of gene annotation, for example through text-mining, has been developed and we wish to test its efficacy. Let us also assume that the previous versions of the GO are worse at the task of enrichment analysis than later versions (as is arguably the case with GDS1962 and *angiogenesis*). We could then combine the novel annotations with previous versions of the GO annotations and observe if key terms rose or fell in significance, and if the new results more closely resemble current results. If so, then we have evidence that the new method of gene annotation is at least as correct as the efforts of existing curators. This would be a powerful new tool for the development of future automated annotation methods.

Similar methods to the ones described here were used in an analysis of a long-term annotation initiative in which the authors examined the impact of the new annotations on standard enrichment analyses [20]. As with our results, they found that the new annotations significantly increased the number of enriched terms, many of which were not present at all before the annotation efforts. Their results are an example of the divergent behavior we would expect from high annotation activity.

## Conclusions

In this framework, we have a quantitative way to examine the GO and its annotations in the context of real-world applications. We have demonstrated its ability to shed light on the evolution of the GO over time, to separately quantify changes in ontology structure and annotation composition, and test the performance of the GO in key applications. Our framework is flexible enough to address many questions facing GO developers and annotators and can be applied across disparate regions of the GO, multiple species, and various enrichment analyses. The methodology presented here should become a valuable tool in the development of novel annotation algorithms and many other applications.

## Methods

To identify a list of genes that were overexpressed in the GDS1962 dataset, we first filtered out microarray probes whose maximum value across samples was less than the median probe value across the entire dataset. We then took the natural log of each probe value. To identify differentially-expressed genes, we used an independent T-test comparing the glioblastoma sample against the control. The final list of differentially expressed genes included those whose Bonferroni-corrected p-value was significant (p < 0.05). We used an enrichment analysis based on Fisher’s exact test for the glioblastoma samples [21]. In the Fisher’s exact test, we used only terms that were annotated to at least 3 and no more than 500 genes.

## Authors' contributions

AS, BG, and EC conceived of the studies; EC and SL performed the analyses; EC, SL and AS wrote the manuscript. All authors read and approved the final manuscript.

## Competing Interests

The authors declare that they have no competing interests.
